# Unilateral Pedicle Screw Fixation is Associated with Reduced Cost and Similar Outcomes in Selected Patients Undergoing Minimally Invasive Transforaminal Lumbar Interbody Fusion for L4-5 Degenerative Spondylolisthesis

**DOI:** 10.7759/cureus.249

**Published:** 2015-02-10

**Authors:** Philip Eliades, Jason P Rahal, Daniel B Herrick, Brian M Corliss, Ron Riesenburger, Steven Hwang, James T Kryzanski

**Affiliations:** 1 Department of Neurosurgery, Tufts University School of Medicine; 2 Wellman Center for Photomedicine and Department of Dermatology, Massachusetts General Hospital/ Harvard Medical School; 3 Department of Neurosurgery, Tufts Medical Center; 4 School of Medicine, Tufts University; 5 Department of Neurosurgery, University of Florida Health

**Keywords:** degenerative spondylolisthesis, transforaminal lumbar interbody fusion, tlif, cost

## Abstract

**Study design:**

Retrospective study of 24 patients who underwent either a bilateral or unilateral TLIF procedure for the treatment of degenerative spondylolisthesis.

**Objective:**

To analyze differences in cost and outcome between patients undergoing minimally invasive transforaminal lumbar interbody fusion (mi-TLIF) with unilateral or bilateral pedicle screw fixation for L4-5 degenerative spondylolisthesis.

**Summary of background data:**

Lumbar fusion surgeries, including the TLIF procedure, have been shown to be an effective treatment for leg and low back pain caused by degenerative spondylolisthesis. Some studies have shown TLIF surgeries to be cost-effective, but there is still a paucity of data and no consensus. Unilateral TLIFs can provide the same benefits as bilateral TLIFs, but come with additional benefits of a less invasive surgery.

**Methods:**

We retrospectively analyzed a consecutive series of patients with L4-5 degenerative stenosis and spondylolisthesis who either received a unilateral or bilateral mi-TLIF, paying particular attention to hospital cost and clinical outcome. Of the 33 patients eligible for analysis, we were able to obtain appropriate clinical and radiographic follow-up data on 24 patients (72.7%), 14 patients who underwent unilateral fixation, and 10 patients who underwent bilateral fixation.

**Results:**

The cohorts were similar with regard to age, comorbidities, and demographics. Most patients reported good or excellent results, and there were no significant differences between the cohorts with regard to clinical outcome. There was one interbody graft extrusion in the unilateral cohort that required explantation, but no other hardware failures. Hospital cost was significantly lower in the unilateral cohort, and hardware savings accounted for only part of the difference.

**Conclusion:**

Unilateral pedicle screw fixation is an acceptable surgical strategy in patients with stable L4-5 degenerative spondylolisthesis undergoing mi-TLIF. In our series, unilateral fixation led to significant hospital cost savings without compromising clinical or radiographic outcomes.

## Introduction

Given the significant portion of United States healthcare spending currently utilized for spinal care and the impetus to contain cost, it is clear that cost-effectiveness of treatments will come under increasing scrutiny. Lumbar fusion procedures, in particular, have received much attention given their expense and strikingly increased utilization over the past two decades [1-2]. From 1992 to 2003, the percentage of Medicare spine surgery spending used for fusion increased from 14% to nearly 50% [1]. Over that period, however, it has become clear that lumbar fusion is clinically effective for patients with common but disabling disorders, such as lumbar spinal stenosis with degenerative spondylolisthesis [3-7]. In fact, prospective trials have consistently shown lumbar fusion to be superior to laminectomy alone in these patients [8-9]. A significant proportion of the cost of lumbar fusion is due to hardware and graft expense; yet there is no consensus on the constructs necessary to achieve satisfactory results. Therefore, it is worthwhile to explore cost reduction through simplifying fusion constructs as long as clinical outcomes are not affected.

Transforaminal lumbar interbody fusion was first described in the early 1980s [10-11], and has produced positive clinical outcomes for the treatment spinal stenosis and degenerative spondylolisthesis [3-7]. Minimally invasive transforaminal lumbar interbody fusion (mi-TLIF) is a less invasive variant of this technique that has grown significantly in popularity and has been described in detail [12-18]. With mi-TLIF, the spinal decompression, interbody arthrodesis, intertransverse arthrodesis, and unilateral pedicle screw fixation may be performed through a single, small, paramedian muscle-splitting exposure that results in reduced blood loss and hospital stay when compared to open TLIF [5, 7, 12, 17-20]. Contralateral pedicle screws may then be placed through an additional paramedian incision, or percutaneously. Unlike open TLIF, bilateral pedicle screw placement in mi-TLIF requires a second, distinct exposure. Therefore, unilateral instrumentation is an attractive option, as it would reduce operative time, instrumentation use, and tissue damage in these patients by avoiding an additional exposure.

Studies comparing unilateral to bilateral instrumentation in TLIF have reported mixed results. Some reports have shown no significant difference in clinical or radiographic outcome, while others have noted poorer clinical outcomes and increased hardware failure in the unilateral cohort [19-21]. Therefore, the appropriateness of unilateral fixation is still unclear. Thorough evaluation of the technique must take into account clinical outcome with appropriate follow-up. The short-term advantages of unilateral fixation would quickly be eclipsed if patients required additional surgery more frequently or had poorer outcomes compared with bilateral pedicle screw fixation. In addition, while there are obvious cost savings to reduced hardware use, there are likely additional savings in reduced operative time, although the magnitude is unknown.

In this study, we seek to address these questions by comparing hospital cost and outcome between patients having mi-TLIF with unilateral or bilateral pedicle screw fixation. We elected to examine patients with L4-5 spinal stenosis and spondylolisthesis since it is a common and generally accepted indication for fusion where surgical results have been favorable. In this study, we present, for the first time, an algorithm for selecting the type of fixation based upon the degree of preoperative instability. Additionally, we quantify the total hospital cost difference between patients undergoing surgery with unilateral or bilateral fixation, which also has not been previously reported.

## Materials and methods

### Cohort selection

We retrospectively reviewed our database of patients undergoing mi-TLIF between 2008 and 2011. We identified 33 patients who had either a unilateral or a bilateral mi-TLIF for L4-5 spinal stenosis and degenerative spondylolisthesis over that period. All patients presented with symptoms of nerve root entrapment and/or neurogenic claudication. Hospital and clinic records, including postoperative phone questionnaires (conducted by an assistant independent of the surgical team), and radiographic studies were reviewed. Hospital cost data was obtained from the Tufts Medical Center billing department. Patients were excluded from the study if they were lost to follow-up. Of the 33 patients, nine were lost to follow-up, leaving a final cohort of 24 patients: 14 with unilateral mi-TLIF and 10 with bilateral mi-TLIF. One of the nine patients lost to follow-up died of unrelated causes, and the other eight could not be contacted.

### Surgical technique

All procedures were performed by the same surgeon (JTK) between 2008 and 2011; informed consent, verbal and written, was obtained from each patient prior to their surgery. A paramedian incision was made over the plane between the longissimus and multifidus components of the sacrospinalis muscle, typically 35-40 mm off midline. A Wiltse paraspinal approach was then used and the Shadowline retractor system (CareFusion, San Diego, CA) maintained exposure [22-23]. A complete facetectomy was performed on the operative side; the contralateral decompression was performed by working medially within the canal. The removed bone was morselized for later use as locally harvested autograft. The disc was entered between the exiting and traversing nerve roots and interbody fusion performed using a CoRoent PEEK structural interbody device (NuVasive, San Diego, CA) packed with a mixture of iliac crest bone marrow and Formagraft (NuVasive, San Diego, CA). Additional Formagraft and morselized autograft was added to the disc space adjacent to the structural device. Next, the pedicle screw fixation was performed using the SpheRx pedicle screw system (NuVasive, San Diego, CA) under fluoroscopic and electromyogram (EMG) monitoring using the NeuroVision system (NuVasive, San Diego, CA). The transverse processes of L4 and L5 were then decorticated and the remainder of the Formagraft and locally harvested autograft was added to the intertransverse space. Finally, the surgical site was copiously irrigated and closed in consecutive layers. In patients with bilateral instrumentation, a second paramedian incision was performed on the contralateral side, and an additional pedicle screw fixation and intertransverse bone graft placement were performed through an additional Wiltse paraspinal exposure.

### Instrumentation algorithm

Patients received either unilateral or bilateral pedicle screw fixation based on radiographic criteria. Unilateral fixation was performed if preoperative dynamic radiographs demonstrated Grade I spondylolisthesis and less than 3 mm of dynamic movement between flexion and extension. One patient in the unilateral cohort had abnormal anatomy that precluded bilateral screw placement. This case will be discussed further in the results and discussion sections.

### Radiographic outcome

Fusion and instrumentation status was assessed by radiographic follow-up. Our standard of practice in the last several years has been to evaluate for fusion primarily with lateral flexion-extension X-ray films, and before that with lumbar computed tomography (CT). Criterion for arthrodesis in patients with follow-up CT scans was presence of trabecular bony bridging in either the intertransverse or interbody spaces. Criteria for arthrodesis in patients with X-ray follow-up included absence of instrumentation pullout or failure, the absence of dynamic translation or angulation on lateral flexion-extension views, and evidence of bony bridging. These fusion criteria are very similar to those used recently by Xue, et al. [21].

### Clinical outcome

Postoperative clinical outcomes were assessed via phone questionnaire, in addition to a review of hospital and clinic notes. The phone interview consisted of a questionnaire that contained a modified version of a scale (Table 1) suggested by Prolo, et al. [24-25]. This questionnaire assessed the surgery’s effect on the patient’s pain status, daily functional capabilities, employment status, and medication needs for residual back pain. Regarding the question about employment status, the ability of a patient to fully participate in desired retirement activities was considered equivalent to working. Patients were also questioned on their subjective level of satisfaction with the surgery, having the options ‘unsatisfied,’ ‘satisfied’ and ‘very satisfied.’

**Table 1:** Modified Prolo Scale *Retirement activities considered equivalent to occupation/work for retired patients. Abbreviations: NSAIDs - nonsteroidal anti-inflammatory drugs.

Score	Description
Pain	
P1	Much worse
P2	Worse
P3	No change
P4	Better
P5	Resolved
Functional Status	
F1	Total incapacity
F2	Can do activities at home
F3	Activities outside home w/ limitation
F4	Limitation w/ strenuous activities
F5	Able to do everything
Economic Status*	
E1	Total incapacity
E2	No gainful occupation
E3	Able to work but not at previous occupation
E4	Working at previous occupation on part-time or limited status
E5	Able to work at previous occupation without restrictions
Medication	
M1	>10 hydrocodone tablets or equivalent
M2	6–9 hydrocodone tablets or equivalent
M3	3–5 hydrocodone tablets or equivalent
M4	Regular NSAIDs or occasional hydrocodone
M5	None or occasional hydrocodone

### Statistical analysis

Statistical analysis was performed with JMP Statistical Discovery Software (SAS Institute, Inc., Cary, NC). Patients who underwent unilateral mi-TLIF were compared to those who underwent bilateral mi-TLIF for gender, comorbidities, clinical outcome grade, and postoperative satisfaction level using Fisher’s exact test. Patient age and length of stay in the hospital were compared between cohorts using Student’s *t*-test. A difference in Prolo score was tested with Wilcoxon signed-rank test, and surgical cost data was analyzed with both the Student’s *t*-test and Wilcoxon signed-rank test. All data is presented as mean ± standard deviation or median [interquartile range (IQR)]. P <0.05 was considered significant.

## Results

**Figure 1:**
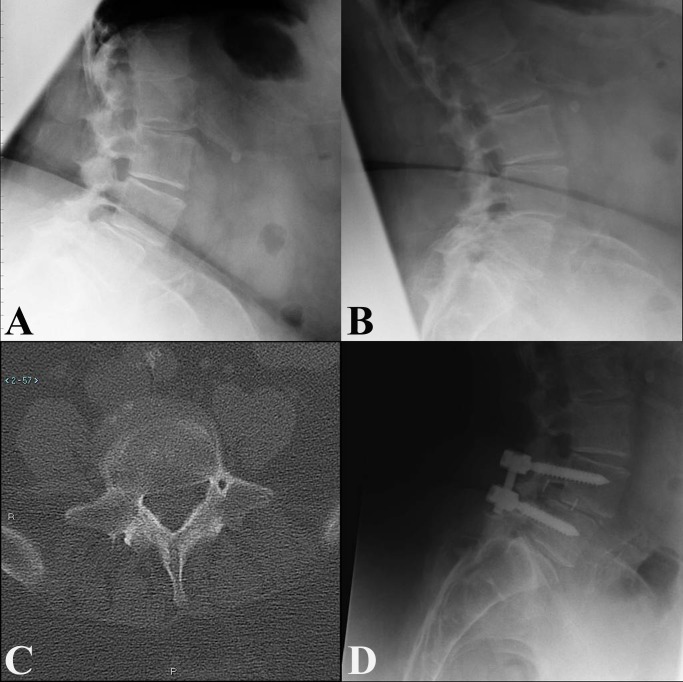
Interbody Graft Extrusion in a Unilaterally-Fixated Patient with an Abnormal Pedicle (A, B) Preoperative flexion (A) and extension (B) radiographs demonstrating anterolisthesis which increases >3mm in flexion. (C) Axial computed tomography through L5 demonstrating a vascular or neural structure within the left L5 pedicle.  (D) 3-month follow-up lateral radiograph demonstrating posterior graft migration.

Of 24 patients, 14 underwent unilateral mi-TLIF, and 10 underwent bilateral mi-TLIF. The patient demographics and comorbidities are provided in Table 2. There were no statistically significant differences between the two cohorts with regards to age or comorbidity. The average length of stay was 4.3 ± 2.1 days for the unilateral cohort, and 4.2 ± 1.0 days (P = 0.90) for the bilateral cohort. One patient in the unilateral cohort suffered postoperative atrial fibrillation that resulted in an extended length of stay of 10 days, an outlier that increased the average length of stay by 0.5 days. This case was also the only perioperative complication in the cohort; no patients in either cohort experienced postoperative radiculitis, venous thrombosis, pneumonia, myocardial infarction, cerebrospinal fluid leak, or surgical site infection. One patient in the unilateral cohort had interbody graft extrusion several months postoperatively requiring explantation. Notably, this patient was considered unstable given the findings of significant translation and angulation on preoperative dynamic imaging studies (Figures 1A, 1B). Bilateral instrumentation was not performed in this case because of a dysmorphic L5 pedicle that appeared to contain a neural or vascular structure (Figure 1C). On three-month follow-up, radiography demonstrated posterior migration of the graft (Figure 1D), which was subsequently explanted.

**Table 2:** Patient Characteristics, by Treatment Group Abbreviations: CAD - Coronary Artery Disease; HIV - Human Immunodeficiency Virus; MI - Myocardial Infarction; SD - Standard Deviation.

	Pedicle Screw Fixation		
	Unilateral (n=14)	Bilateral (n=10)	P-value
Age (years), ± SD	66.8 ± 6.5	62.5 ± 7.9	0.18
Female, n (%)	10 (71.4)	9 (90.0)	0.36
Comorbidities, n (%)			
Diabetes	1 (7.1)	2 (20.0)	0.55
HIV	1 (7.1)	0 (0.0)	1.00
Smoking history	5 (35.7)	1 (10.0)	0.34
CAD	2 (14.3)	0 (0.0)	0.49
History of MI	1 (7.1)	0 (0.0)	1.00
Rheumatoid arthritis	0 (0.0)	1 (10.0)	0.42

### Surgical cost

Hospital cost was significantly different between unilateral mi-TLIF and bilateral mi-TLIF and is presented in Table 3. The median hospital bill for a unilateral mi-TLIF was $48,867 [$42,496 - $49,986], significantly less than the median bill for a bilateral mi-TLIF, which was $67,610 [$58,171 - $70,858] (P = 0.004). Likewise, the mean cost for the unilateral cohort was significantly less than that for the bilateral cohort ($49,918 ± $17,579 vs. $63,554 ± $9,874; P = 0.04).

**Table 3:** Surgical Cost Data, by Treatment Group Abbreviations: IQR - Interquartile Range; SD - Standard Deviation.

	Pedicle Screw Fixation		
	Unilateral (n=14)	Bilateral (n=10)	P-value
Total hospital bill, median [IQR]	$48,867 [$42,496 - $49,986]	$67,610 [$58,171 - $70,858]	0.004
Total hospital bill, mean ± SD	$49,918 ± $17,579	$63,554 ± $9,874	0.04

### Radiographic outcome

Mean radiographic follow-up was 16.6 ± 9 months. Nineteen patients (79%; 11 from the unilateral cohort, and eight from the bilateral cohort) had a minimum of 12 months of radiographic follow-up. Of these patients, all demonstrated evidence of fusion (100%) on follow-up imaging (seven AP/lateral X-rays, eight flexion-extension X-rays, and four CTs).

### Clinical outcome

Mean clinical follow-up was 28.1 ± 12.5 months. Clinical outcomes are reported in Table 4. The median Prolo score among the patients receiving a unilateral TLIF was 19 (15.75 - 20), one point higher than the median score of 18 (15.75 - 19.25) among the patients receiving a bilateral TLIF (P = 0.61). The patient distributions among the different outcome grades were very similar between the unilateral and bilateral cohorts (P = 0.96). In the unilateral cohort, 92.9% of patients and 90% of patients in the bilateral cohort scored ‘good’ or ‘excellent’ for clinical outcome. Patient satisfaction was also similar between the unilateral and bilateral instrumentation cohorts (P = 0.66), with no patient in either cohort being ‘unsatisfied.’ Other than the patient who required interbody graft explantation, no patient required any additional lumbar surgery in the clinical follow-up period.

**Table 4:** Postoperative Clinical Outcome Grade, Modified Prolo Scores, and Satisfaction, by Treatment Group *Determined by Modified Prolo Score – Excellent, 18-20; Good, 15-17; Fair, 11-14; Poor, ≤10. Abbreviations: IQR - Interquartile Range.

	Pedicle Screw Fixation		
	Unilateral (n=14)	Bilateral (n=10)	P-value
Clinical outcome grade*, n (%)			
Excellent	9 (64.3)	6 (60.0)	0.96
Good	4 (28.6)	3 (30.0)	
Fair	1 (7.1)	1 (10.0)	
Poor	0 (0.0)	0 (0.0)	
Prolo score, median [IQR]	19 [15.75 - 20]	18 [15.75 - 19.25]	0.61
Level of satisfaction, n (%)			
Very satisfied	11 (78.6)	7 (70.0)	0.66
Satisfied	3 (21.4)	3 (30.0)	
Unsatisfied	0 (0.0)	0 (0.0)	

## Discussion

The new standard in the United States healthcare system is for treatments to be judged against cost by determining cost-effectiveness or cost-utility. Cost-utility analysis is a subcategory of cost-effectiveness analyses where the effect measured is the gain in quality of life from undergoing a procedure, or the quality-adjusted life years (QALYs). In this way, different procedures and treatments across medical fields can be compared to one another.

Instrumented spinal fusions have inconsistently met common cost-effectiveness thresholds. Adogwa, et al. published data from a prospective study of 45 patients showing that TLIF surgeries resulted in cost/QALYs gained of close to $42,000 [3]. This finding supports an earlier study that demonstrated cost/QALYs gained of $51,000 [26]. These numbers fall in the neighborhood of $50,000, which is often considered to be a cost-effective threshold [3]. However, earlier studies have shown the costs to exceed what would be considered cost-effective. A prospective cohort study using randomized and observational cohorts determined the surgical treatment of degenerative spondylolisthesis to be beneficial, but at a cost of $115,600 per QALY gained [27]. Similarly, two other studies performed with a societal perspective acknowledge that surgical treatment is favored, but concluded that instrumented fusion, compared to non-instrumented fusion, was much too costly for the relatively small incremental gain in outcome [28-29]. With some studies evaluating TLIFs to be cost-effective in the treatment of pain due to degenerative spondylolisthesis and others considering instrumented fusions to be cost-ineffective, it is acknowledged that patient selection has a large effect on cost/QALYs, and that a relative paucity of cost studies regarding TLIFs remains [3, 29].

It is also clear from the previous analyses, and intuitively, that reducing the cost of a procedure improves cost-effectiveness as long as outcome is not affected. In our study, we saw significant cost-savings associated with unilateral instrumentation in patients with stable spondylolisthesis and no significant difference in clinical or radiographic outcome. The cost savings was of a significant magnitude, and only a minority of the difference was explained by reduced hardware cost. 

The difference in median costs between unilateral and bilateral fusions was more statistically significant than the difference between mean costs. We believe the median values better represent the difference since there was an outlier in the unilateral cohort whose hospital bill ($106,267) was more than double the next most expensive surgery in that cohort, seemingly because of a lengthy hospital stay due to postoperative atrial fibrillation. Only a fraction of the observed cost difference between the unilateral and bilateral TLIFs can be directly attributed to hardware savings. Based on instrumentation costs for our hospital provided by the supplier, NuVasive, the additional hardware used in a bilateral procedure increased the cost by roughly $2,700-$3,500 depending on the year and specific hardware used, which means only about 15-19% of the difference in median cost is accounted for by hardware. Other sources of cost difference include operating room services, anesthesia, sterile supply, recovery room services, and pharmacy. Unfortunately, the hospital’s billing department was unable to provide enough cost data from these sources for us to perform more detailed analyses between the two cohorts. Based on findings in aforementioned studies, we also expected the unilateral cohort to have a shorter length of stay contributing to decreased cost; however, our study does not support this claim as both our cohorts have very similar length of stays. We suspect this may be due to the older study populations of both cohorts, which included many Medicare patients who are required three days of hospitalization before transitioning to rehabilitation facilities. 

There are several limitations to this study. Firstly, the retrospective nature of the analysis is clearly a weakness, particularly with regard to outcomes. It was because of this that we limited our study to patients with single level L4-5 spinal stenosis and degenerative spondylolisthesis who presented with nerve root entrapment and/or neurogenic claudication. These patients are known to have good postoperative outcomes, and indeed our cohort overall had good clinical results and a high satisfaction rate. We do not feel the effectiveness of surgery for this condition needs to be re-proven, instead the primary goals of our outcome analysis were to evaluate for parity between the cohorts and identify clinical or hardware failures. Another limitation of outcome analysis lies in the fundamental difference between the two cohorts related to the degree of preoperative instability. This is due to our algorithm, which bases fixation on the amount of preoperative dynamic instability, as previously described. However, it has not been demonstrated to our knowledge that preoperative instability correlates with the severity of presentation. Given the good outcomes seen in both cohorts we feel it is likely that any such difference is small and does not seem to affect the postoperative results. Lastly, our study size was smaller than others comparing TLIF with unilateral or bilateral fixation because we narrowed our criteria to one specific pathology.

Our findings support an algorithm for instrumentation placement that considers the degree of instability on dynamic imaging of patients with degenerative spondylolisthesis. This has not been previously described. Recent studies comparing unilateral to bilateral instrumentation in TLIF have met with conflicting results: Duncan, et al. reported increased cage migration in patients with unilateral fixation while Xue, et al. showed no clinical or radiographic differences [20-21]. These studies randomized patients into either fixation group without regard to stability and also included diverse indications for fusion with degenerative spondylolisthesis in only a minority of patients. Aoki, et al. randomized patients with degenerative spondylolisthesis to unilateral or bilateral fixation and found poorer clinical results in the unilateral group [19]. A biomechanical study using a finite-element computer simulation model demonstrated that for certain types of intervertebral cage placements unilateral pedicle screw fixation may not provide adequate stability during axial rotation and lateral bending [30]; the clinical translation of these simulated findings is questionable, especially when preoperative stability is used for operative planning. The one hardware failure in our series occurred in a patient who met our preoperative instability criteria for a bilateral fusion, but received unilateral instrumentation due to an anomalous pedicle. Therefore, it appears that bilateral instrumentation is necessary for patients with significant instability and this may help explain poorer outcomes seen in randomized studies. Our study supports the hypothesis that unilateral fixation is acceptable for some but not all patients with degenerative spondylolisthesis. Our series showed no other hardware failures or obvious pseudoarthroses, but postoperative evaluation of bony fusion presents challenges and was another limitation of our study. Ideally, CT scans showing bridging trabecular bone are obtained, but given the increasingly recognized risk of medical radiation, we do not routinely obtain postoperative lumbar CT scans in patients doing well clinically and showing no signs of hardware loosening or instability on plain films.

Though our analysis demonstrates the cost savings of unilateral instrumentation, an additional way to limit cost would be to identify a subset of patients with spinal stenosis and degenerative spondylolisthesis who are at a low risk for post-laminectomy instability and would do well without fusion. Existing prospective studies comparing laminectomy alone to laminectomy with fusion did not take into account the degree of instability [8-9]. Therefore, it would be useful to examine this and other factors in the future to see if such a patient subset could be recognized preoperatively.

## Conclusions

In conclusion, in selected patients with L4-5 degenerative spondylolisthesis mi-TLIF with unilateral instrumentation provides similar outcome with reduced cost compared to mi-TLIF with bilateral instrumentation. Although unilateral fixation is not appropriate for every patient, we can also conclude, as evidenced by our findings, that a preoperative algorithm for choosing appropriate unilateral fixation candidates helps preserve excellent clinical outcomes.
